# Effects of high viscosity bone cement percutaneous vertebroplasty on pain, bone specific alkaline phosphatase, Type-I collage cross-linked-telopeptide and Boneglaprotein levels in patients with osteoporotic vertebral compression fractures

**DOI:** 10.12669/pjms.38.6.5828

**Published:** 2022

**Authors:** Guangcheng Zhang, Juan Zhu, Qing Zhang, Jiazhu Tang, Mingjun Nie

**Affiliations:** 1 Guangcheng Zhang, Dept. of Orthopedics, Affiliated Hospital of Jiangsu University, Zhenjiang, Jiangsu Province, 212000, P.R. China; 2 Juan Zhu Dept. of Anesthesiology, Affiliated Hospital of Jiangsu University, Zhenjiang, Jiangsu Province, 212000, P.R. China; 3 Qing Zhang, Dept. of Orthopedics, Affiliated Hospital of Jiangsu University, Zhenjiang, Jiangsu Province, 212000, P.R. China; 4 Jiazhu Tang, Dept. of Orthopedics, Affiliated Hospital of Jiangsu University, Zhenjiang, Jiangsu Province, 212000, P.R. China; 5 Mingjun Nie, Dept. of Orthopedics, Affiliated Hospital of Jiangsu University, Zhenjiang, Jiangsu Province, 212000, P.R. China

**Keywords:** High viscosity bone cement, Percutaneous vertebroplasty, Osteoporotic vertebral compression fracture, Complication, Bone metabolism

## Abstract

**Objectives:**

To investigate the effects of percutaneous vertebroplasty with high viscosity bone cement on pain and the levels of bone specific alkaline phosphatase (BALP), Type-I collagen cross-linked telopeptide (CTX) and serum osteocalcin (BGP) in patients with osteoporotic vertebral compression fractures.

**Methods:**

The medical records of patients with osteoporotic vertebral compression fractures treated in our hospital from February 2020 to February 2021 were selected after retrospective analysis. Patients (43) who received low viscosity bone cement percutaneous vertebroplasty comprised Group-I, and patients (56) who received high viscosity bone cement percutaneous vertebroplasty, comprised Group-II of the study. The occurrence of bone cement leakage, pain (VAS scores), BALP, CTX and BGP were compared and analyzed between the two groups.

**Results:**

The incidence of bone cement leakage in Group-II was 16.28%, lower than 3.57% in Group-I (P<0.05). The visual analogue scale (VAS) scores of patients in Group-II were lower than those in Group-I at one and three months after the surgery (P<0.05). The levels of BALP and BGP in Group-II were higher than those in Group-I three months after the surgery (P<0.05), and CTX was lower than those in Group-I (P<0.05).

**Conclusions:**

Percutaneous vertebroplasty with high viscosity bone cement in the treatment of osteoporotic vertebral compression fractures can reduce the incidence of bone cement leakage and help to further reduce pain and improve bone metabolism.

## INTRODUCTION

Osteoporotic vertebral compression fractures are commonly seen in the elderly. It has an incidence of 23% in these patients.^1^ and can seriously reduce their lumbar function. At present, treatment of osteoporotic vertebral compression fractures includes conservative treatment, such as bed rest, braces, oral drugs, etc., and surgical treatment methods. Although the pain symptoms of patients can be relieved to a certain extent, they need to stay in bed for a long time, which can increase the risk of complications such as accumulated pneumonia and pressure ulcer, as well as seriously reduce patients’ quality of life.^2^,^3^

In recent years, minimally invasive surgery gradually became the first choice for osteoporotic vertebral compression fractures. Studies show that it can effectively stabilize the fracture, promote the vertebral height, restore the spinal stability, effectively relieve the patient’s pain, and promote the improvement of lumbar function. Percutaneous vertebroplasty is one of the most common surgical methods used for treatment of osteoporotic vertebral compression fractures. It involves making a percutaneous puncture, flowed by establishment of pedicle working channel and the injection of bone cement, using imaging equipment for monitoring.

Percutaneous vertebroplasty can promote the increase of vertebral strength and rapidly alleviate pain symptoms.^4^ When treating patients with osteoporotic vertebral compression fractures by percutaneous vertebroplasty, the selection of the correct type of bone cement is crucial. In the past, low viscosity bone cement was mainly selected clinically. However, it is characterized by increased fluidity and fast diffusion, and easily leaks from fractures and bone wall defects, leading to complications related to bone cement leakage.^5^,^6^ In contrast, after modulation, high viscosity bone cement has no liquid phase, can be injected for a longer time, has low polymerization temperature, significant analgesic effect and reduced leakage rate.^7^ Therefore, in recent years, our hospital has mostly selected high viscosity bone cement percutaneous vertebroplasty for the treatment of osteoporotic vertebral compression fractures. The aim of this study was to further analyze the efficiency of high viscosity bone cement for percutaneous vertebroplasty.

## METHODS

Clinical records of 99 patients (37 males and 62 females) with osteoporotic vertebral compression fractures, treated by bone cement percutaneous vertebroplasty from February 2020 to February 2021, were retrospectively analyzed. The ethics committee of Jiangsu University affiliated Hospital approved this study (No. KY2021K1002, November 19^th^ 2021-). According to the viscosity of bone cement used, the selected subjects were divided into low viscosity group (Group-I) and high viscosity group (Group-II).

### Inclusion criteria:

• Met the diagnostic criteria of osteoporotic vertebral compression fracture under CT and MRI;^8^
• No spinal cord injury and nerve compression;
• Voluntary surgical treatment;
• Complete medical records;
• Fully cooperated with the treatment and gave informed consent.

### Exclusion criteria:

• Other serious basic diseases, organ dysfunction and malignant tumor;
• Pathological vertebral fracture;
• Skin infection around the puncture point;
• Cognitive and mental disorders.

All the patients underwent percutaneous vertebroplasty. Briefly, patient was brought to prone position to keep the abdomen suspended, and ECG monitoring was performed. C-arm X-ray machine was used to accurately locate the target vertebral body of the surgical patient, and the body surface of the pedicle was projected. The surgical site of the patient was routinely disinfected and covered with towel, and then anesthetized with 2% lidocaine local infiltration. After performing anesthesia, a longitudinal small incision was made at the puncture point. Then, the operation of the puncture of the trocar puncture on the oblique side of the pedicle was completed under the assistance of the C arm X-ray machine. After the bone surface was penetrated, the direction of the puncture needle was adjusted, so that it was parallel to the endplate, and toward the middle line, entering the vertebral body. Before reaching the 1/3 of the vertebral body, the vertebral body was punctured by the same method. After the puncture, the puncture pillow core was pulled out, and the bone cement (low viscosity bone cement, OSTEOPAL®V, Heraeus, Germany; high viscosity bone cement, Confidence Spinal Cement System®, Teknimed, France) was put into the bone cement needle barrel, and injected slowly into the vertebral body with the assistance of C-arm X-ray machine (if there was bone cement leakage during the injection, the injection was stopped immediately and treated accordingly). After observing that the injected bone cement was well distributed and completely hardened, the puncture needle was pulled out, and the wound was wrapped with sterile dressing. The vital signs of patients were closely observed 12 hours after operation, patients were encouraged to move after 24 hours to reduce complications such as thrombosis, and intravenous antibiotics were given routinely once to prevent infection.

### The following clinical indexes were calculated within three months after the operation:

• The incidence of bone cement leakage in intervertebral disc, spinal canal, paravertebral soft tissue and paravertebral vein.
• The pain of the patient on the day of admission, assessed using VAS scoring till system,^9^ on a scale of zero to ten (0: painless, 10: unbearable severe pain).
• Serum levels of BALP, CTX and BGP on the day of admission. Briefly, 4ml fasting venous blood samples were collected and analyzed by Roche automatic modular serum analysis system. Pain and BALP, CTX and BGP levels one and three months after operation were further followed up by outpatient visits, telephone or wechat.

SPSS22.0 was used to analyze and process the collected data. [n(%)] was used to represent the non-grade count data, and the test method is *x*^2^;(*x̅*± s) was used to represent measurement data. Normal distribution was analyzed by t-test, and rank sum test was used for not-normal distribution. *P*< 0.05 indicated statistically significant results.

## RESULTS

A total of 99 patients (37 males and 62 females) met the inclusion criteria. The age ranged from 54 to 76 years, with an average of (64.75±5.94) years. Medical records indicated 34 cases of single segment thoracic fracture, 42 cases of single segment lumbar fracture and 23 cases of double segment fracture. Of 99 patients, 43 received low viscosity bone cement percutaneous vertebroplasty and 56 received high viscosity bone cement percutaneous vertebroplasty.

There was no significant difference in basic clinical characteristics between the two groups (P>0.05) (Table-I). The incidence of bone cement leakage in Group-II was 3.57%, significantly lower than 16.28% in Group-I (P<0.05) (Table-II). On the day of the admission, there was no significant difference in VAS score between the two groups (P>0.05). At one and three months after operation, the VAS scores of the two groups were lower than those on the day of admission, and the VAS scores of patients in Group-II were significantly lower than in Group-I (P<0.05) (Table-III). On the day of the admission, there was no significant difference in the levels of BALP, CTX and BGP between the two groups (P>0.05). At three months after the operation, the levels of BALP and BGP in the two groups were higher than those on the admission day, and the levels of CTX in Group-II were lower than those on the admission day(P<0.05). The levels of BALP and BGP in Group-II were higher than those in Group-I at three months after operation (P<0.05), and CTX levels were significantly lower than those in Group-I (P<0.05), (Table-IV).

**Table I:** Comparison of general information between the two groups.

Group	n	Gender (M/F)	Age (year)	Fracture segment (n)		

				Single-segment thoracic spine fracture	Single-segment lumbar fracture	Two-segment fracture
I group	43	15/28	64.39±5.54	11	17	9
II group	56	22/34	65.02±6.27	17	25	14
x^2^/t	-	0.201	0.515	0.762		
P	-	0.654	0.608	0.683		

**Table II:** Comparison of the incidence of bone cement leakage between the two groups [n(%)].

Group	n	Bone cement leakage				Total

		Intervertebral disc leakage	Intraspinal leakage	Leakage of paravertebral soft tissue	Leak in the paravertebral veins	
I group	43	3 (6.98)	2 (4.65)	1 (2.33)	1 (2.33)	7 (16.28)
II group	56	1 (1.79)	1 (1.79)	0 (0.00)	0 (0.00)	2 (3.57)
*x* ^2^	-	-	-	-	-	4.753
P	-	-	-	-	-	0.029

**Table III:** Comparison of pain between the two groups (*x̅*±s, points).

Group (n)	Admission day	1 month after operation	3 months after surgery
I group (n=43)	8.09±0.89	2.60±0.88^a^	1.42±0.50^a^
II group (n=56)	8.14±0.84	1.95±0.44^ab^	1.03±0.33^ab^
t	0.284	4.500	4.358
P	0.777	<0.001	<0.001

***Note***: Compared with this group before treatment,

a: P<0.05, compared with I regimen treatment group, b: P<0.05.

**Table IV:** Comparison of the levels of BALP, CTX and BGP between the two groups (*x̅*±s).

Group (n)	BALP (U/L)		*t*	*P*	CTX (mg/L)		*t*	*P*	BGP (ug/L)		*t*	*P*
		
	Admission day	3 months after surgery			Admission day	3 months after surgery			Admission day	Treatment completion date		
I group (n=43)	38.30±4.14	43.63±4.12	24.203	<0.001	436.35±26.96	417.56±25.72	39.480	<0.001	5.44±0.91	6.18±0.66	11.054	0.010
II group (n=56)	38.12±3.97	51.89±4.40	48.327	<0.001	429.41±25.34	343.28±24.78	560.091	<0.001	5.43±0.81	7.28±0.59	31.280	<0.001
t	0.216	9.528	-	-	1.313	14.542	-	-	0.077	8.672	-	-
P	0.829	<0.001	-	-	0.192	<0.001	-	-	0.939	<0.001	-	-

## DISCUSSION

Our results showed that the incidence of bone cement leakage in Group-II was 3.57%, significantly lower than 16.28% in Group-I (P<0.05), suggesting that percutaneous vertebroplasty with high viscosity bone cement can further reduce the incidence of bone cement leakage in patients with osteoporotic vertebral compression fractures. Kong M et al.^10^ Compared and analyzed the occurrence of bone cement leakage during percutaneous vertebroplasty with high viscosity and low viscosity bone cement and found that the bone cement leakage rate in the high viscosity bone cement group was significantly lower, which was consistent with the results of this study. Bone cement leakage is one of the common complications of percutaneous vertebroplasty, and its incidence depends mainly on the viscosity of bone cement. After modulation, high viscosity bone cement is already in a dough like high viscosity state with no liquid phase. It has low polymerization temperature, which allows to control the injection direction and amount, more accurate injection into the target vertebral body, and as a result, reduces risk of bone cement leakage.^11^

Pain is the most obvious symptom of osteoporotic vertebral compression fracture. Study by Wang WF et al.^12^ and others show that the VAS score of patients with vertebral compression fracture is significantly lower after surgical treatment. In this study, the VAS scores at one and three months after operation in Group-II were lower than those in Group-I (P<0.05), suggesting that percutaneous vertebroplasty with high viscosity bone cement is more helpful to relieve pain in the treatment of osteoporotic vertebral compression fractures. High viscosity bone cement can instantly reach the state of high viscosity, prolong the injection time, facilitate the adjustment of injection position during the operation, make the bone cement evenly dispersed on the vertebral body and smoothly enter the anterior column. It promotes more balanced enhancement on both sides of the vertebral body, enhances the biological performance of the vertebral body, further consolidates the fracture and strengthens the vertebral body. These characteristics allow effective alleviation of the pain symptoms in patients.^13^ Many clinical studies have shown that the recovery of lumbar function in patients with vertebral compression fracture is closely related to bone metabolism. BALP, CTX and BGP are common bone metabolism indicators. The synthesis and secretion of BALP and BGP occurs mainly in osteoblasts, and may, therefore, reflect the state of bone formation. CTX is a common bone resorption marker which can reflect the state of bone resorption.^14^

A study by Wang M et al.^15^ compared and analyzed the levels of BALP, CTX and BGP in patients with osteoporotic thoracolumbar fracture and normal population, and showed that BALP and BGP decreased significantly and CTX increased in patients with osteoporotic thoracolumbar fracture. The results of our study showed that the levels of BALP and BGP in Group-II were higher than those in Group-I at three months after operation (P<0.05), and CTX was lower than those in Group-I (P<0.05), suggesting that percutaneous vertebroplasty with high viscosity bone cement in patients with osteoporotic vertebral compression fracture can further improve the postoperative bone metabolism. Percutaneous vertebroplasty with high viscosity bone cement can make the bone cement evenly distributed in the vertebral body, restore the stability of the injured vertebral body, achieve balanced compressive capacity on both sides of the vertebral body, promote early healing of the fracture and make the fracture more stable. The number of osteoclasts decreases and promotes the formation of osteoblasts, thus improving bone metabolism.^16^

### Limitations of this study:

Only 99 patients admitted to our hospital in the recent one year were selected. Moreover, the number of observation indicators is limited. Relatively short follow-up observation (up to three months after the operation) has high subjectivity, which may make the conclusions one-sided and limited.

## CONCLUSION

In the treatment of patients with osteoporotic vertebral compression fracture, the selection of high viscosity bone cement for percutaneous vertebroplasty can reduce the leakage rate of bone cement, and help to further alleviate the pain and improve the indexes of bone metabolism.We hope this study can provide reference for the treatment of fracture patients

### Authors’ contributions:

**GZ:** Conceived and designed the study. **JZ, QZ, JT & MN:** Collected the data and performed the analysis. **GZ:** Was involved in the writing of the manuscript and is responsible for the integrity of the study. All authors have read and approved the final manuscript.
